# Spatial regulation of NSUN2-mediated tRNA m^5^C installation in cognitive function

**DOI:** 10.1093/nar/gkae1169

**Published:** 2024-12-14

**Authors:** Yulia Gonskikh, Christian Tirrito, Praneeth Bommisetti, Maria Saraí Mendoza-Figueroa, Julian Stoute, Joshua Kim, Qin Wang, Yuanquan Song, Kathy Fange Liu

**Affiliations:** Department of Biochemistry and Biophysics, Perelman School of Medicine, University of Pennsylvania, Philadelphia, PA 19104, USA; Biology Graduate Group, University of Pennsylvania, School of Arts and Sciences, Philadelphia, PA 19104, USA; The Raymond G. Perelman Center for Cellular and Molecular Therapeutics, The Children's Hospital of Philadelphia, Philadelphia, PA 19104, USA; Department of Biochemistry and Biophysics, Perelman School of Medicine, University of Pennsylvania, Philadelphia, PA 19104, USA; Department of Biochemistry and Biophysics, Perelman School of Medicine, University of Pennsylvania, Philadelphia, PA 19104, USA; Department of Biochemistry and Biophysics, Perelman School of Medicine, University of Pennsylvania, Philadelphia, PA 19104, USA; The Raymond G. Perelman Center for Cellular and Molecular Therapeutics, The Children's Hospital of Philadelphia, Philadelphia, PA 19104, USA; The Raymond G. Perelman Center for Cellular and Molecular Therapeutics, The Children's Hospital of Philadelphia, Philadelphia, PA 19104, USA; The Raymond G. Perelman Center for Cellular and Molecular Therapeutics, The Children's Hospital of Philadelphia, Philadelphia, PA 19104, USA; Department of Pathology and Laboratory Medicine, University of Pennsylvania, Philadelphia, PA 19104, USA; Department of Biochemistry and Biophysics, Perelman School of Medicine, University of Pennsylvania, Philadelphia, PA 19104, USA; Graduate Group in Biochemistry and Molecular Biophysics, Perelman School of Medicine, University of Pennsylvania, Philadelphia, PA 19104, USA; Abramson Family Cancer Research Institute, University of Pennsylvania Perelman School of Medicine, Philadelphia, PA 19104, USA; Penn Institute for RNA Innovation, University of Pennsylvania, Philadelphia, PA 19104, USA; Penn Center for Genome Integrity, University of Pennsylvania, Philadelphia, PA 19104, USA

## Abstract

Enzyme-mediated modifications of tRNA, such as 5-methylcytosine (m^5^C) installed by nuclear-enriched NOP2/Sun RNA methyltransferase 2 (NSUN2), play a critical role in neuronal development and function. However, our understanding of these modifications' spatial installation and biological functions remains incomplete. In this study, we demonstrate that a nucleoplasm-localized G679R NSUN2 mutant, linked to intellectual disability, diminishes NSUN2-mediated tRNA m^5^C in human cell lines and *Drosophila*. Our findings indicate that inability of G679R-NSUN2 to install m^5^C is primarily attributed to its reduced binding to tRNA rather than its nucleoplasmic localization. Conversely, an NSUN2 variant lacking an internal intrinsically disordered region (ΔIDR-NSUN2) can install ∼80% m^5^C within the nucleoplasm. Furthermore, we show that tRNA m^5^C levels are positively correlated to cognitive performance in *Drosophila*, where expressing G679R-NSUN2 leads to the most severe social behavioral deficits while expressing ΔIDR-NSUN2 results in less pronounced deficits. This work illuminates the molecular mechanism underlying G679R disease mutation in cognitive function and offers valuable insights into the significance of the cellular localization of m^5^C installation on tRNA for neuronal function.

## Graphical Abstract

**Graphical Abstract:**
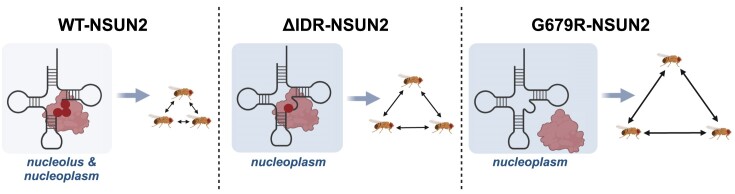

## Introduction

RNA modifications represent an important layer of gene expression regulation. Chemical modifications affect RNA structure, RNA stability, base pairing, binding to protein partners and overall RNA function (1). Recent data demonstrate that RNA and RNA-modifying enzymes are implicated in various biological processes, and their dysregulation is connected to numerous human diseases (2).

The transfer RNAs (tRNAs) are the most extensively modified RNA species (3). tRNAs are transcribed as precursors by Polymerase III and undergo a series of sequential maturation steps: removing 5′ and 3′ tRNA trailer sequences, intron splicing for intron-containing pre-tRNA, 3′-CAA tail addition, as well as installment of various base and sugar modifications by tRNA modifying enzymes. Furthermore, all steps of tRNA processing are spatially ordered. In eukaryotes, tRNA transcription and early steps of tRNA biogenesis occur in the nucleolus (4–6), while later tRNA biogenesis steps occur in the nucleoplasm. Then, tRNA is exported into the cytoplasm by exportin-t (7), where the mature tRNAs are aminoacylated and become ready to participate in protein synthesis. Some of the tRNA modifications serve as quality control of tRNA biogenesis (8), some promote correct tRNA folding, stabilize tRNA tertiary structure (9), ensure tRNA stability, while other tRNA modifications regulate decoding during protein synthesis (10).

One of the most abundant tRNA modifications is 5-methylcytidine (m^5^C). Most of the m^5^C modifications in cytoplasmic tRNAs are installed by two enzymes: SAM-dependent methyltransferases DNMT2 and NOP2/Sun RNA methyltransferase 2 (NSUN2). While DNMT2 only methylates C38 of tRNA^Asp^, tRNA^Val,^ and tRNA^Gly^ (11–13), NSUN2 methylates up to 80% of the cytoplasmic tRNAs (14) as well as several mitochondrial tRNAs (mtRNAs) (15,16). NSUN2 is primarily located in the nucleolus (17), while DNMT2 is primarily located in the nucleoplasm. However, whether the nucleolar localization of NSUN2 is important for NSUN2 function in methylating tRNA is unknown.

Several studies demonstrate that NSUN2-mediated m^5^C increases tRNA stability and promotes either global protein synthesis or introduces a bias toward codon-specific translation. Knockout of NCL1/TRM4, the yeast NSUN2 ortholog, is sensitive to translational inhibitor paromomycin (18). Chan and colleagues showed that m^5^C at position 34 of yeast tRNA^Leu(CCA)^ installed by the NSUN2 ortholog enhances the translation of the UUG-rich mRNAs (19). In mice, neuronal *Nsun2* knockout leads to a reduction in tRNA^Gly^ level and consequent reduction in the translation of the Gly-rich proteins (20). Additionally, Nsun2 deficiency in mice is linked to the accumulation of 5′ tRNA fragments derived from hypomodified tRNAs and the reduction of overall protein synthesis (14).

The function of NSUN2 is linked to neurodevelopment in several organisms. In mice, *Nsun2* knockout (KO) causes a reduction in body and brain size (21), as well as a reduction in the brain/body ratio (20). In *Nsun2* KO mice, reduced levels of tRNA^Gly^ cause global proteome changes and, as a result, impair neurotransmission and contextual fear memory (20). Deletion of the NSUN2 ortholog in *Drosophila melanogaster* cause short-term memory deficits that could be rescued by re-expression of the wild type protein in neurons (22).

Similarly, NSUN2 function is connected to intellectual disability in humans. Several mutations in the *NSUN2* gene are identified as the primary cause of autosomal-recessive forms of intellectual disability (22–24). Among the reported NSUN2 mutations, all identified cases except one are nonsense mutations. Here, we focus on the only missense mutant of G679R-NSUN2 that causes intellectual disabilities in humans (23). We reveal the molecular mechanism of G679R-NSUN2 dysfunction using a set of cellular and biochemical assays. Utilizing *Drosophila* as a model organism, we demonstrate that G679R-NSUN2 cannot methylate tRNAs, leading to an impaired social behavior phenotype. Furthermore, using an NSUN2 version with the largest intrinsically disordered region (IDR) deleted, we study the significance of nucleolar localization for m^5^C installment on tRNAs and cognitive function in *Drosophila*.

## Materials and methods

### Mammalian cell culture and construction of *NSUN2* knockout cell lines

HEK293T cells were cultured in DMEM (Gibco 11995–065) with 10% FBS (Cytiva SH30910.03) and 1% penicillin/streptomycin (Gibco 15140–122) in a humidified incubator with 5% CO2 at 37°C. For transient transfection, Lipofectamine 2000 (Thermofisher 11 668 019) or jetOPTIMUS (Polyplus 101 000 025) were used according to the manufacturer's instructions. To create NSUN2 knockout cell lines, two pX459 plasmids expressing gRNA5 and gRNA6 were transfected into HEK293T cells. Twenty-four hours after transfection, cells were treated with 2 μg/ml Puromycin (Gibco A11138-03) for 48 h. Followed by 24-h recovery, cells were seeded as single cells in 96 well-plates. Two weeks later, the isolated colonies were screened by the genotyping polymerase chain reaction (PCR) using primers hNSUN2_KO5-6_for and hNSUN2_KO5-6_rev. Further validation was conducted by Western blotting with an anti-NSUN2 antibody (Invitrogen PA5-115674). The sequences of sgRNAs and genotyping PCR primers are listed in Supplementary Table S1.

### Plasmid construction

To express sgRNA targeting NSUN2 gene in human cells, two pairs of DNA oligos were individually cloned (gRNA5_for, gRNA5_rev for gRNA5 and gRNA6_for, gRNA6_rev for gRNA6) into pX459 vector digested with BbsI (NEB R3539S).

NSUN2 open reading frame was amplified from HeLa cDNA and confirmed by Sanger sequencing. G679R mutation was introduced by site-directed mutagenesis PCR (25) using primers NSUN2_G679R_for and NSUN2_G679R_rev and confirmed by Sanger Sequencing. IDR truncation was introduced by fusion PCR with the primers NSUN2_ΔIDR_for and containing NSUN2_ΔIDR_rev and confirmed by colony PCR. For transient transfection, NSUN2 open reading frame was cloned into the pPB vector, using NSUN2_Mfe1_for and NSUN2_Xho1_rev primers using MfeI and XhoI cutting sites. For protein purification, NSUN2 was subcloned into PMALc2x vector (Addgene Plasmid #75 286) containing MBP tag at the N-terminus using the forward primer containing the sequence of the TEV cleavage site (NSUN2_TEV_EcoRI_for) and reverse primer containing His-tag sequence (NSUN2_6His_SalI_rev). For expression of the NSUN2 into flies’ neurons, NSUN2 was subcloned from the pPB plasmids into the pACU2 plasmid (Addgene #31 223) using PPB_XhoI_for and NSUN2_XbaI_rev primers. The sequences of PCR primers are listed in Supplementary Table S1.

### Fluorescence immunostaining

Immunostaining analysis was performed as previously described (26). In short, HEK293T NSUN2 KO cells were transfected with 1 μg of pPB-NSUN2 plasmid in a 6-well plate. Twenty-four hours after transfection, the cells were washed with PBS and fixed using 4% paraformaldehyde prepared in DMEM media at 37°C for 15 min. Then, the cells were washed twice by PBST (PBS with 0.05% Tween-20) and permeabilized by 0.5% Triton prepared in PBS at room temperature for 20 min. After permeabilization, the cells were washed with PBST, blocked in 1% BSA in PBST at room temperature for 30 min and then incubated overnight at 4°C in the Fluor-conjugated anti-NSUN2 antibody (Proteintech CL488-66580). After overnight incubation, cells were washed with PBST and stained with DAPI by incubation in 0.5 mg/mL DAPI for 1 min. After four PBST washes, an antifade reagent (Invitrogen) was used to mount the slides. The images were taken using a Leica TCS SP8 confocal microscope. The quantification of NSUN2 intensity was performed using Fiji software. To represent NSUN2 nucleolar localization, the NSUN2 intensity in the fibrillarin-rich region was divided by its intensity in the nucleoplasm of the same cell.

For immunohistochemistry in flies, third instar larvae were dissected and their brains were subjected to immunostaining. The following antibodies were used: mouse anti-FLAG antibody (F3156, 1:1000, Sigma) and fluorescence-conjugated secondary antibodies (1:1000, Jackson ImmunoResearch). Adult fly brains were dissected and immunostained with mouse anti-FasII antibody (1:100, DHSB, 1D4).

### Quantitative analysis of the m^5^C level in small RNA

To assess m^5^C level in tRNAs, small RNA (<200 nt long) was extracted with the RNA Clean and Concentrate kit (Zymo Research R1013) according to the manual. Then 150 ng of the purified RNA were digested and dephosphorylated to single nucleosides using Nucleoside Digestion Mix (New England Biolabs M0649S) at 37°C for 2 h. After digestion, samples were injected into liquid chromatography-mass spectrometry (LC-MS/MS) in two replicates as described before (27). The nucleosides were quantified using retention time and the nucleoside-to-base ion mass transitions of 268.1 to 136.1 (A), 284.1 to 152.1 (G), 258.1 to 126.1 (m^5^C and m^3^C), 247 to 115 (D), 258 to 112 (Cm), 282.1 to 150.1 (m^1^A), 286.1 to 154.1 (ac4C), 312 to 180 (m^2^_2_G), 336 to 204 (i^6^A) and 298.1 to 166.1 (m^1^G). Quantifications of m^5^C, C and G were performed by converting the peak area from the LC-MS/MS to fmol using the standard curve obtained from pure nucleoside standards running with the same batch of samples under otherwise identical conditions. The percentage ratio of each modification to C or G was used to compare the modification levels across samples.

### Isolation of mitochondria and mtRNAs

To assess the status of m^5^C modification of mtRNAs after expression of WT- and ΔIDR-NSUN2, mClover-WT-NSUN2 and mClover-ΔIDR-NSUN2, the mClover-NSUN2 constructs were transiently expressed in NSUN2 KO HEK239T cells. Twenty-four hours after transfection, the transfection efficiency and expression level were estimated by measuring the mClover signal using a Guava Easy 5HT instrument (Luminex 0500–4005). The cells with high and comparable mClover signals were subjected to mitochondria extraction as previously described (16). In short, detached cells were washed twice with PBS and resuspended in homogenization buffer (10 mM Tris-HCl pH 7.4, 0.6 M mannitol, 0.1 mM EDTA, and protease inhibitors). Cells were homogenized with 10 strokes in a glass Dounce homogenizer. Homogenates were first centrifuged at 1000 g for 10 min to remove cell debris and nuclei, and then for 10 min at 10 000 g to pellet the mitochondria. The obtained mitochondrial pellet was either resuspended in Laemmli buffer for the Western blotting or subjected to total RNA, followed by small RNA extraction for mass spectrometry analysis. Efficient cellular fractionation was confirmed by Western blotting with Tom20 (Abcam, 186 734) and Vinculin (Cell Signaling, 4650) as markers for mitochondrial or cytoplasmic fractions, respectively.

### Protein purification

To purify recombinant MBP-NSUN2 proteins, PMALc2x plasmids were transformed into Escherichia coli strain BL21 for expression. The bacteria were cultured at 37°C at 200 rpm, and when the OD_600_ of the culture was ∼0.8, the protein expression was induced by 1 mM IPTG for 17 h at 16°C. The cell pellets were then resuspended in lysis buffer (25 mM Tris pH 7.5, 500 mM NaCl) and sonicated. Lysates were cleared by centrifugation at 12 000 rpm for 30 min and were loaded to a Ni-NTA column (QIAGEN). The lysis buffer supplemented with 50 mM imidazole was used as a washing buffer and the same buffer supplemented with 500 mM imidazole was used for elution. The crude products were further purified by size-exclusion chromatography with a Superdex S75 column (GE) in storage buffer (25 mM Tris-HCl pH 8, 50 mM NH_4_Ac, 5% glycerol).

### *In vitro* methylation with recombinant proteins

To evaluate the catalytic activity of MBP-WT-NSUN2 and MBP-G679R-NSUN2, we performed an *in vitro* methylation assay with recombinant proteins on 28 nt long RNA oligo resembling part of tRNA^Gly^ (Supplementary Table S1). Prior reaction, RNA oligo was denatured for 2 min at 90°C and placed on ice. Each reaction in 20 μl total volume contained 200 ng of RNA oligo, 20 mM Tris pH 8.0, 50 mM NH_4_Ac, 2 mM DTT, 1 mM SAM and 1 μM MBP-NSUN2. The reaction was carried out at 37°C for the indicated time. Then samples were subjected to RNA extraction with the Clean and Concentrate kit (Zymo Research R1013). Recovered RNA was subjected to RNA digestion, dephosphorylation and LS-MS/MS as described above. The data were fit to the one phase exponential association equation using Prism software.

### Preparation of nuclear lysates

To assess the catalytical activity of ΔIDR-NSUN2 *in vitro*, mClover-ΔIDR-NSUN2 and mClover-WT-NSUN2 were transiently expressed in NSUN2 KO HEK239T cells. Twenty-four hours after transfection, the transfection efficiency and expression level were estimated by measuring the mClover signal using a Guava Easy 5HT instrument (Luminex 0500–4005). The cells with high and comparable mClover signals were used to prepare nuclear lysates as previously described (28). For that, detached cells were washed twice with PBS, resuspended in cytoplasmic extract buffer (10 mM HEPES pH 7.6, 1.5 mM MgCl2, 10 mM KCl, 0.15% NP-40) and incubated on ice for 5 min. Next, lysates were fractionated into cytoplasmic and nuclear fractions via centrifugation at 4000 g for 5 min. The obtained nuclear pellet was resuspended in equal volume of nuclear extract buffer (20 mM HEPES pH 7.6, 1.5 mM MgCl2, 420 mM NaCl, 0.2 mM EDTA, 20% glycerol) and subjected to three freeze-thaw cycles: 15 min at −80°C followed by thawing at 37°C with vortexing for 1 min in between cycles. After that, the nuclear lysates were clarified by centrifugation at 15 000 g for 10 min. Lysates concentrations was estimated using Bradford reagent. The lysates were diluted to the same concentration using nuclear extract buffer. Comparable NSUN2 concentration in the nuclear extracts was confirmed using Western blotting, where Lamin B1 (Proteintech, 660 095) served as a loading control.

### *In vitro* methylation with nuclear lysates

To compare the catalytic activity of WT- and ΔIDR-NSUN2, we performed *in vitro* methylation assays with nuclear extracts obtained from cells transiently expressing mClover-ΔIDR-NSUN2 and mClover-WT-NSUN2. *In vitro* methylation reaction was performed on 5′-biotynylated 28 nt long RNA oligo resembling part of tRNA^Gly^ (Supplementary Table S1), which was denatured for 2 min at 90°C prior reaction. Each reaction in 200 μl total volume contained 200 pmol of pre-denatured RNA oligo, 20 mM Tris pH 8.0, 50 mM NH_4_Ac, 2 mM DTT, 1 mM SAM, RNase inhibitor and 45 μl of 3.5 μg/μl nuclear extract. The reaction was carried out at 37°C for 16 h. Then streptavidin beads (Invitrogen, 65–001) were used to purify RNA probes, following the instructions from the manufacturer. Afterward, RNA on the beads were digested and dephosphorylated to the single nucleotides using Nucleoside Digestion Mix (New England Biolabs, M0649S) at 37°C and 1400 rpm for 5 h and were analyzed by LS-MS/MS as described above.

### *In vitro* transcription

To create a DNA template for *in vitro* transcription of tRNA^Gly^, two overlapping 64 and 62 nt oligos (tRNA_Gly_for containing T7 promotor sequence and tRNA_Gly_rev) were annealed and extended. For that, 2 μM of each primer were combined with 0.5 mM each dNTP, 1 μl of Taq Polymerase in a total volume of 50 μl and subjected to the following program: initial denaturation for 5 min at 98°C, 10 cycles of denaturation for 30 s at 98°C, annealing for 1 min at 52°C, elongation for 1 min at 72°C, and final elongation for 5 min at 72°C. Extended primers were then used as a template for PCR with the shorter primers tRNA_Gly_for2 and tRNA_Gly_rev2. The obtained PCR product was purified with Phenol Chloroform Isoamyl alcohol (Sigma, P3803) and used as a transcription template. A transcription reaction was performed using the HiScribe T7 High Yield RNA Synthesis Kit (NEB, E2040S). In the total volume of 20 μl reaction contained 1× reaction buffer, 1 μg of the purified PCR template, 10 mM ATP, 10 mM CTP, 10 mM GTP, 2.5 mM UTP, 1 μl of ^32^P-UTP (PerkinElmer), 1 μl RNase inhibitor RNaseOUT (Thermofisher, 10 777 019) and 2 μl T7 RNA polymerase. Transcription was carried out for 15 h at 37°C. To separate RNA product from unincorporated UTP, the reaction was passed through the G-25 Sephadex columns (Cytiva, 27 532 501). To evaluate the quality of the transcribed tRNA, we run the transcription product (1 μl of 1:50 dilution in water) on 8% PAA 7 M Urea gel in 1× TBE. After running, the gel was dried for 45 min 80°C under a vacuum and exposed to the phosphorimager screen overnight.

### Electrophoretic mobility shift assay (EMSA)

To measure the binding of NSUN2 to the tRNA substrate, we used *in vitro* transcribed ^32^P-tRNA^Gly^ (prepared as described above). In total volume of 7 μl, each reaction contained 20 mM Tris pH 8.0, 50 mM NH_4_Ac, 154 ng of PolyU, 3 U of RNase inhibitor RNase RNaseOUT (Thermofisher 10 777 019), 0.5 μl of 1:50 diluted in water *in vitro* transcribed ^32^P-tRNA^Gly^ and indicated amount of the NSUN2-MBP protein. The reaction was incubated for 30 min at 37°C. After incubation, the entire reaction in 1× TBE Sample buffer (Invitrogen LC6678) was separated on 6% PAA 1× TBE non-denaturing gel. After running, the gel was vacuum-dried for 45 min at 80°C and exposed to the phosphorimager screen. After overnight exposure, the screen was scanned using a phosphor imager Typhon FLA 9500, and signals were quantified using the software ImageJ.

### Fly stocks

The *Canton-S* wild type and *elav-Gal4* stocks were obtained from Bloomington *Drosophila* Stock Center. To generate the UAS-FLAG-WT-NSUN2, UAS-FLAG-ΔIDR-NSUN2 and UAS-FLAG-G679R-NSUN2 stocks, the coding sequences were cloned into the pACU2 vector, then injected into fly embryos via φC31 mediated site-specific insertion (Rainbow Transgenic Flies, Inc). To generate the *Nsun2* knockout flies, 5′-GGCCTGACGATCGGTGCGCTTGG-3′ was selected as the target. For gRNA synthesis, the sense oligo (5′-5Phos/CTTCGGCCTGACGATCGGTGCGCT-3′) and antisense oligo (5′-5Phos/AAACAGCGCACCGATCGTCAGGCC-3′) were annealed and ligated into a BbsI-digested pU6-BbsI chiRNA vector. The gRNA construct was then injected into *vas-Cas9* flies (Rainbow Transgenic Flies, Inc), and the resultant potential mutants were screened and verified by PCR (29).

### Social space assay

Social space assay was performed as described (30). In brief, 6–8 days post-eclosion, ∼30–40 males were aspirated by mouth pipetting (to avoid confounding behavioral effects from carbon dioxide exposure) into the social space arena: a triangular chamber with ∼7 inch sides and a ∼1 cm depth. The flies were given 20 min to freely investigate the arena. Images were then captured. The distance between any of the two flies was measured by Image J and normalized to the average body length of flies in pixels (22). The calculation of the social space index is based on the values obtained in the histogram representations of the social distance. It equals the percentage of flies in the first bin (0–2 number of body length) minus the percentage of flies in the second bin (2–4 number of body length) (31).

### Negative geotaxis assay

Around 10–15 flies were gently transferred to an empty vial and were habituated for at least 1 min. The total number of flies in each vial was then recorded to account for any flies that may have escaped during transferring. The flies were then tapped to the bottom and were given 10 s to climb up. Immediately after the 10 s, the number of flies that passed a marked height of 8 cm was recorded. The ‘average pass rate’ was generated by obtaining the percentage of flies that successfully climbed past the 8 cm line, divided by the total number of flies in that vial.

### tRNA purification

To assess the status of m^5^C modification of individual tRNAs after expression of WT- and ΔIDR-NSUN2, we transiently expressed mClover-WT-NSUN2 and mClover-ΔIDR-NSUN2 in *NSUN2* KO HEK239T cells. Twenty-four hours after transfection, the transfection efficiency and expression level were estimated by measuring the clover signal using a Guava Easy 5HT instrument (Luminex, 0500–4005). The cells with high and comparable clover signals were subjected to small RNA extraction. To perform tRNA purification, 500 pmol of 5′ biotinylated DNA oligo complementary to the tRNA targets (tRNAPD_Leu CAA, tRNAPD_Gly GCC, tRNAPD_Asp GUC and tRNAPD_Glu UUC) were incubated with the 45 μl of the 10 mg/mL pre-washed Streptavidin Dynabeads M-280 (Invitrogen 11206D) in 100 μl of 10 mM Tris pH 7.5 overnight at 4°C with rotation. After overnight incubation, the beads were washed three times with 10 mM Tris pH 7.5 to remove unbound DNA. To denature tRNAs, 500 ng of small RNA in 50 μl water were incubated at 80°C for 10 min and combined with 50 μl of pre-warmed 2× TMA buffer (20 mM Tris pH 7.5, 1.8 M Tetramethylammonium chloride, 0.2 mM EDTA). The obtained mixture was added to the 15 μg of dry DNA-bound beads, incubated at 65°C for 10 min, gradually cooled down from 65 to 37°C, and incubated for 30 min at 37°C. During incubations, the samples were flicked every 5 min to minimize sedimentation of the beads. The beads were washed five times with 10 mM Tris pH 7.5 to remove unbound RNA. For LC-MS/MS analysis the purified tRNA were digested and dephosphorylated on the beads. For Northern blotting, the tRNA was eluted in 2× RNA loading dye at 90°C for 5 min.

### Northern blotting

To test the efficiency of the tRNA affinity purification, 5 ng of the input samples and 20% of the purified tRNA were denatured in 1× RNA loading dye at 95°C for 2 min and separated on denaturing polyacrylamide gels (7 M urea). Subsequently, gels were stained with Ethidium Bromide, and RNA was transferred onto nylon membranes (Amersham Hybond N+, GE Healthcare) at 400 mA for 45 min. After UV cross-link, membranes were hybridized to a ^32^P-5′-end-labeled DNA probe complementary to the tRNA targets or 5*S* rRNA overnight at 42°C, washed and exposed to a phosphor imager screen.

## Results

### G679R-NSUN2 leads to tRNA m^5^C hypomethylation

**Figure 1.**
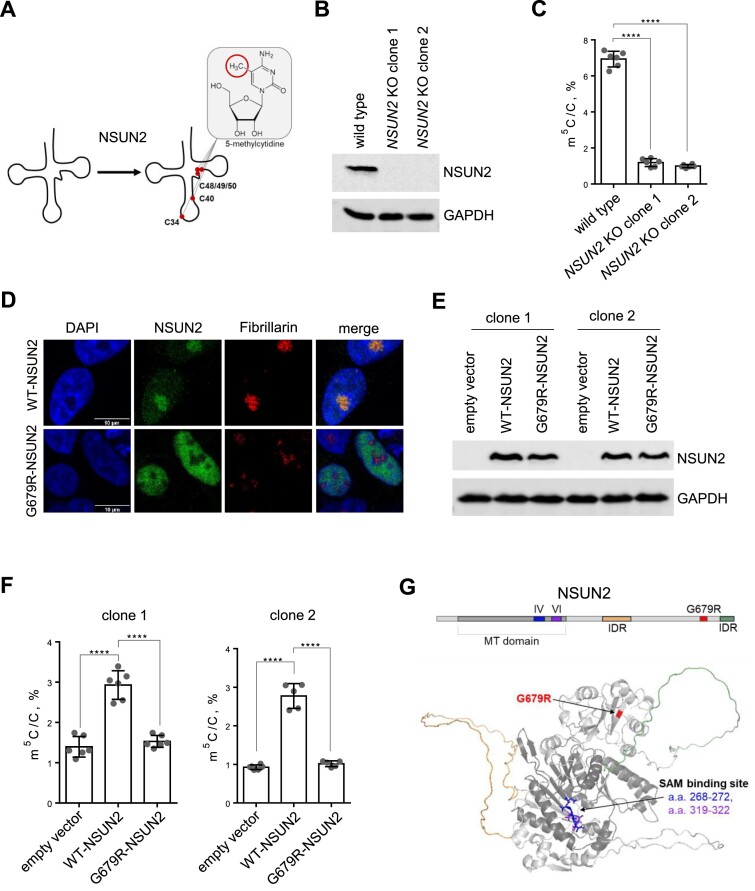
G679R-NSUN2 is located in the nucleoplasm and leads to diminished m^5^C in tRNAs. (**A**) Wild type NSUN2 installs m^5^C on cytoplasmic tRNAs at positions C48, C49, C50, C40 and C34. (**B**) Western blots with anti-NSUN2 and anti-GAPDH antibodies showing NSUN2 expression in *NSUN2* knockout clones (*NSUN2* KO). GAPDH serves as a loading control. (**C**) LC-MS/MS quantification of m^5^C in small RNAs (<200 nt) isolated from parental (wild type) or *NSUN2* KO HEK293T clones. Three biological replicates represent every sample, each was measured in two technical replicates. The two-tailored *t*-test is used to calculate the *P*-value (*P* < 0.0001). Error bars represent mean ± S.D. (**D**) Representative immunofluorescence images with anti-NSUN2 antibody of WT- and G679R-NSUN2 which have been transiently transfected in *NSUN2* KO HEK293T cells. Fibrillarin serves as a nucleolus marker. Nuclei is stained with DAPI. Scale bars, 10 μm. (**E**) Representative western blots with anti-NSUN2 and anti-GAPDH antibodies showing the expression levels of exogenous WT-NSUN2 and G679R-NSUN2, which have been transiently transfected in *NSUN2* KO HEK293T cells. (**F**) LC-MS/MS quantification of m^5^C in <200 nt RNAs isolated from *NSUN2* KO cells transiently expressing empty vector, WT-NSUN2 and G679R-NSUN2. Three biological replicates represent every sample, each was measured in two technical replicates. The two-tailored *t*-test was used to calculate the *P*-value (*P* < 0.0001). Error bars represent mean ± S.D. (**G**) Structural model of human NSUN2 predicted using AlphaFold3. The G679R mutation site, SAM-binding pocket, and IDRs are indicated.

Human NSUN2 installs m^5^C on most cytoplasmic tRNAs in the variable loop (Figure 1A). While most identified NSUN2 mutations causing intellectual disability (22,23) are nonsense mutations, G679R remains the only reported missense mutation of NSUN2. Prior to our study, it was unknown whether the nucleoplasmic G679R-NSUN2 can install m^5^C in tRNA. First, we generated an *NSUN2* KO in HEK293T cell line using CRISPR-Cas9 with two pairs of single guide-RNAs (sgRNAs) targeting exons 8 and 9 of the *NSUN2* gene (Supplementary Figure S1A). We screened for *NSUN2* KO single colonies using PCR on genomic DNA with the primers framing the cut sites (Supplementary Figure S1B, C). Two KO single colonies were used for western blots to verify that NSUN2 expression has diminished (Figure 1B). To assess m^5^C modification level in tRNAs, we performed triple quadruple LC-MS/MS analysis on small RNA (<200 nt) upon *NSUN2* KO. First, the area of the detected m^5^C and C peaks were converted to the molar abundance using the standard curves (Supplementary Figure S2A–D). Then the abundance of m^5^C modification was normalized to the abundance of C nucleosides in the same sample. The results of LC-MS/MS analyses demonstrated a significant reduction of the m^5^C levels in small RNAs (<200 nt) upon *NSUN2* KO in both single colonies (Figure 1C, Supplementary Figure S2E–G); the other abundant tRNA modifications did not show significant differences after *NSUN2* KO (Supplementary Figure S2H). Next, we expressed wild type (WT)-NSUN2 and G679R-NSUN2 in *NSUN2* KO cells. The immunofluorescence imaging results suggest that WT-NSUN2 is enriched in the nucleolus as indicated by the nucleolar marker protein fibrillarin; in contrast, G679R-NSUN2 is mislocalized to the nucleoplasm (Figure 1D), which is consistent with a previous study (23). Next, we confirmed comparable expression level of WT-NSUN2 and G679R-NSUN2 in *NSUN2* KO cells via western blots (Figure 1E). The LS-MS/MS results showed that WT, but not G679R-NSUN2, can restore the m^5^C levels in small RNA, the majority of which are tRNAs (Figure 1F). A crystal structure of human NSUN2 is not yet available; thus, we created AlphaFold3-predicted structure of full-length human NSUN2 (uniport Q08J23-1). The structure shows that G679R is located far from the SAM-binding pocket and active sites of NSUN2 (Figure 1G), thus, it does not explain the catalytic deficiency.

### An NSUN2 truncation variant in the nucleoplasm region partially rescues m^5^C levels in tRNAs

**Figure 2.**
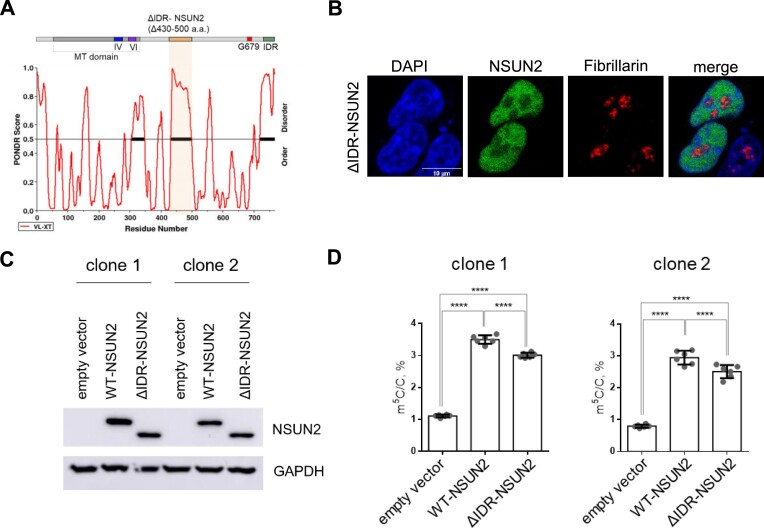
ΔIDR-NSUN2 truncation variant is located in the nucleoplasm and partially rescues the m^5^C levels in tRNAs. (**A**) Structural prediction of NSUN2 using PONDR (natural disordered regions). The largest predicted IDRs are indicated. (**B**) Representative immunofluorescence images with anti-NSUN2 antibody of ΔIDR-NSUN2, which has been transiently transfected in NSUN2 KO HEK293T cells. Fibrillarin serves as a nucleolus marker. Nuclei is stained with DAPI. Scale bars, 10 μm. (**C**) Representative western blots with anti-NSUN2 and anti-GAPDH antibodies showing the expression of exogenous WT-NSUN2, and ΔIDR-NSUN2, which have been transiently transfected in *NSUN2* KO HEK293T cells. (**D**) LC-MS/MS quantification of m^5^C in <200 nt RNAs isolated of *NSUN2* KO cells transiently expressing empty vector, WT-NSUN2 and ΔIDR-NSUN2. Every sample is represented by three biological replicates; each was measured in two technical replicates. The two-tailored *t*-test was used to calculate the *P*-value (*P* < 0.0001). Error bars represent mean ± S.D.

The cellular hypomethylation of m^5^C in tRNA caused by missense G679R-NSUN2 intrigued us to study whether this phenotype is due to G679R’s nucleoplasm localization or its intrinsic defects in catalysis. To understand whether the nucleolar localization of NSUN2 is critical for tRNA m^5^C installation, we aim to design an NSUN2 mutant that mimics the nucleoplasm localization of G679R-NSUN2. Because many protein factors enter the nucleolus via undergoing the biophysical process called liquid-liquid phase separation (LLPS) (32), and IDRs of the proteins are known for promoting LLPS (33), we predicted the disordered regions of NSUN2 using the neural network predictor Predictor Of Naturally Disordered Regions (PONDR). The results suggest that NSUN2 contains three IDRs: region 1 being from amino acids 306–348; region 2 being from amino acids 430–500; and region 3 being from amino acids 719–767. We thus generated an NSUN2 truncation variant with the largest predicted disordered region depleted (ΔIDR-NSUN2, 430–500 amino acids) (Figure 2A). Immunofluorescence imaging results showed that ΔIDR-NSUN2 is mislocalized from the nucleolus to the nucleoplasm which is reminiscent of G679R-NSUN2 (Figure 2B, Supplementary Figure S3A). Next, we re-expressed WT- and ΔIDR-NSUN2 into *NSUN2* KO cells at a comparable level (Figure 2C). The LC-MS/MS results revealed that expression of both, WT-NSUN2 and ΔIDR-NSUN2, significantly increased m^5^C levels but did not affect other tRNA modifications (Figure 2D, Supplementary Figure S3B), suggesting that m^5^C installment on tRNA by NSUN2 can take place outside of the nucleolus. Interestingly, a 20% decrease in tRNA m^5^C level is observed in cells expressing nucleolar-depleted ΔIDR-NSUN2 compared to nucleolar-enriched WT-NSUN2 (Figure 2D).

### G679R’s catalytic defect is due to significantly reduced tRNA-binding affinity

**Figure 3.**
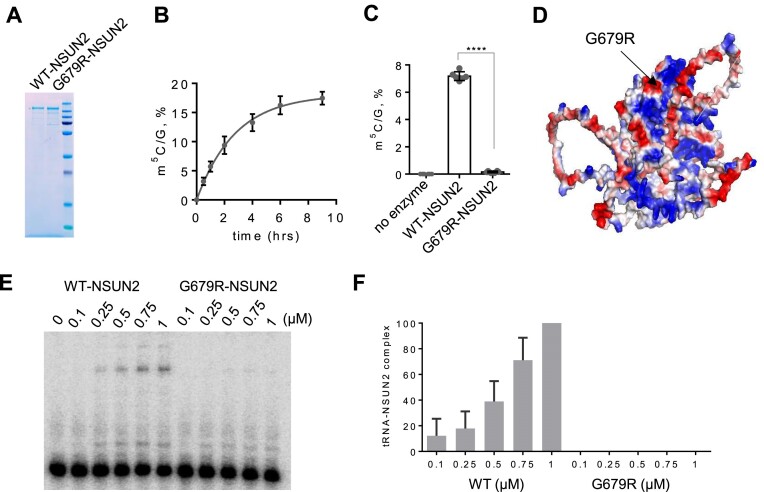
G679R-NSUN2 presents significantly impaired tRNA-binding affinity and catalytic efficiency. (**A**) Sodiumdodecyl sulphate-polyacrylamide gel electrophoresis showing the purity of purified recombinant MBP-WT-NSUN2 and MBP-G679R-NSUN2. (**B**) LC-MS/MS quantification of m^5^C levels in an unmodified RNA probe after *in vitro* methylation reaction with recombinant WT-NSUN2 at different incubation time intervals. Error bars represent mean ± SEM. (**C**) LC-MS/MS quantification of m^5^C levels of RNA probe after *in vitro* methylation reaction with recombinant WT- and G679R-NSUN2 for 1 h. Error bars represent mean ± S.D. The two-tailored t-test is used to calculate the *P*-value (*P*< 0.0001). (**D**) Surface electrostatic potential of human NSUN2 (structure prediction was done with AlphaFold3). (**E**) EMSA showing WT- and G679R-NSUN2 binding to *in vitro* transcribed tRNA**. (F)** Quantification of the EMSAs. *n* = 6. Error bars represent mean ± S.D. n.d., not determined.

As ΔIDR-NSUN2 (nucleoplasmic localization) can restore most m^5^C in small RNAs, the nucleoplasmic localization of G679R may not be causative for the hypomethylation status of the tRNAs. To assess the catalytical activity of NSUN2, we performed *in vitro* methylation reaction with an RNA oligo resembling one NSUN2 substrate, tRNA^Gly^, using recombinant proteins (Figure 3A). The LC-MS/MS results showed that full-length WT-NSUN2 effectively installed m^5^C in this RNA probe (Figure 3B). In contrast to WT-NSUN2, the G679R-NSUN2 variant failed to install m^5^C on the RNA oligo (Figure 3C). We next analyzed the electrostatic potential of NSUN2 using PyMOL. The electrostatic surface map showed that arginine 679 is located in a highly positively charged region of the protein, which might be involved in tRNA binding (Figure 3D). Thus, to determine whether the G679R mutation impairs tRNA binding and subsequently abolishes its catalytic activity, we performed the electrophoretic mobility shift assay (EMSA) using *in vitro* transcribed ^32^P-labeled tRNA^Gly^ (Supplementary Figure S4). While WT-NSUN2 formed a high molecular weight protein/RNA complex in a concentration-dependent manner, G679R-NSUN2 presented almost no protein-RNA complex formation (Figure 3E, F). These results indicate that G679R’s catalytic defect is largely due to the reduced tRNA-binding affinity.

### ΔIDR-NSUN2, but not G679R-NSUN2, partially rescues social behavioral deficits observed in *Drosophila Nsun*2 KO

**Figure 4.**
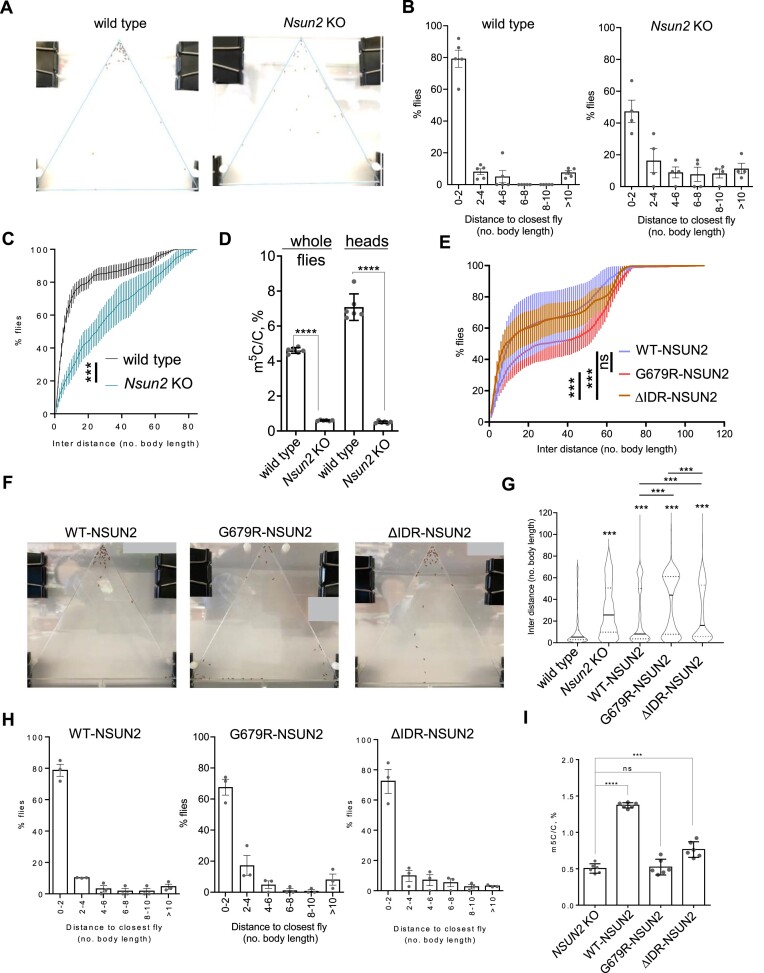
Assess the function of NSUN2-mediated m^5^C installation in tRNA in *Drosophila* social interactions. (**A–C**) In the social space assay, *Nsun2^5-2^* knockout (*Nsun2* KO) flies display disrupted social behavior as revealed by their aberrant distribution in the device and the increased interdistance. (**A**) Representative images showing fly distribution in the social behavior assay. Error bars represent mean ± SEM. (**B**) Histogram of the nearest neighbor distance. (**C**) The cumulative frequency of fly interdistance in the device. Data are analyzed by two-way ANOVA followed by Sidak's test. *n* = 3 groups, with ∼30–40 flies per group. (**D**) LC-MS/MS quantification of m^5^C levels in small RNA (<200 nt) of wild type or *Nsun2* KO flies. RNA is extracted either from the whole flies or flies’ heads. Three biological replicates represent every sample, each was measured in two technical replicates. The two-tailored t-test was used to calculate the *P*-value. Error bars represent mean ± S.D. (**E–H**) Rescue of social interaction defects in *Nsun2* KO by pan-neural overexpression of human NSUN2 transgenes. WT-NSUN2 and ΔIDR-NSUN2 show robust and partial rescue, respectively, while G679R fails to rescue. (**E**) The cumulative frequency of fly interdistance in the device. Data are analyzed by two-way ANOVA followed by Tukey's test. (**F**) Representative images showing fly distribution in the social behavior assay. (**G**) Quantification of the distance between any of the two flies in each trial. Data are analyzed by one-way ANOVA followed by Tukey's test. Each group is compared to every other group. Compared to wild type, all other groups are significantly increased. Compared to WT-NSUN2, G679R-NSUN2 and ΔIDR-NSUN2 exhibit significantly less rescue. (**H**) Histogram of the nearest neighbor distance. *n* = 3–5 groups, with ∼30–40 flies per group. Error bars represent mean ± SEM. (**I**) LC-MS/MS quantification of m^5^C levels in heads’ small RNA (< 200 nt) extracted from *Nsun2* KO flies after pan-neuronal expression of human WT-, G679R- and ΔIDR-NSUN2. Every sample is represented by three biological replicates; each was measured in two technical replicates. Error bars represent mean ± S.D. In all figures: ns stands for not significant. *P < 0.05, ***P < 0.001, ****P < 0.0001.

To determine the biological function of NSUN2-mediated m^5^C installation in tRNAs, we utilized *Drosophila melanogaster* as a model organism. First, we created a knockout of the *Nsun2* fly homolog using the CRISPR system (Supplementary Figure S5A). We generated three alleles, *Nsun2^5-2^* (874 bp deletion and replacement with GACA), *Nsun2^4-3^* (5 bp deletion) and *Nsun2^8-2^* (4 bp insertion). LS-MS/MS results showed that all three alleles drastically reduced m^5^C levels (Supplementary Figure S5B). We thus focus on *Nsun2^5-2^* hereafter and designate it as the *Nsun2* KO. The remaining m^5^C is likely due to an additional methyltransferase Dnmt2 in *Drosophila*. In the negative geotaxis assay, which measures adult fly locomotion, *Nsun2* KO flies displayed drastically reduced capacity to reach a preset climbing threshold, despite their capability to move and fly (Supplementary Figure S5D). Human NSUN2 deficiency has also been linked to autism (34,35). Thus, we evaluated the social behavior of the flies using a social space assay (31), wherein flies were placed in a closed vertical triangular chamber. After 20 min, we measured and compared the distance between individual flies to determine their inclination to social interactions. *Nsun2* KO flies exhibited increased social space, indicated by the increased interdistance compared to wild type flies and the decreased social space index (Figure 4A–C, Supplementary Figure S5E). Decreased social space index results from higher percentage of flies in the 2–4 number of body length and low percentage in the 0–2 number of body length, that is, the flies are more spread out. This reflects social deficits, as seen in flies with loss-of-function of autism-related genes (36–38). LS-MS/MS on the small RNAs (<200 nt) isolated from *Nsun2* KO flies demonstrated a clear reduction of the m^5^C level (Figure 4D). To elucidate if human NSUN2 mutants could rescue the fly social behavioral deficits, we expressed human WT-, G679R- and ΔIDR-NSUN2 in neurons of *Nsun2* KO flies using the pan-neural driver elav-Gal4. We confirmed that all three transgenes are expressed in the fly brain (Supplementary Figure S5F). Expression of WT-NSUN2 mitigated the increased interdistance and decreased social space index (Figure 4E–H, Supplementary Figure S5E). Notably, the disease mutant G679R-NSUN2 exhibited the least effective rescue of the behavioral phenotype. Interestingly, flies expressing ΔIDR-NSUN2 partially rescued the behavioral phenotype but showed a significant difference in the social space assays compared to those expressing WT-NSUN2 (Figure 4F–H). Moreover, WT-NSUN2 is also more effective in rescuing the negative geotaxis behavior compared to the G679R-NSUN2 and ΔIDR-NSUN2 variants (Supplementary Figure S5D). Consistent with our previous results, LS-MS/MS on small RNA isolated from fly heads showed partial rescue of the m^5^C levels after WT-NSUN2 expression. ΔIDR-NSUN2 expression rescues m^5^C levels to a lesser extent than WT-NSUN2 expression (Figure 4I). There are no significant changes in the relative abundance of other tRNA modifications upon *Nsun2* KO, nor after re-expression of WT-, G679R- or ΔIDR-NSUN2 (Supplementary Figure S5G).

### Nucleoplasmic ΔIDR-NSUN2 partially rescues m^5^C of specific tRNA

LC-MS/MS analysis on small RNA (<200 nt) extracted from cells expressing either WT- or ΔIDR-NSUN2 consistently revealed significant differences in m^5^C levels (3.5% for WT-NSUN2 versus 3% for ΔIDR-NSUN2 in clone 1, and 3% for WT-NSUN2 versus 2.5% ΔIDR-NSUN2 in clone 2) (Figure 2D). Similarly, expressing human WT-NSUN2 in the neurons of *Drosophila* revealed that tRNA m^5^C levels are significantly higher in comparison to flies expressing ΔIDR-NSUN2 (1.4% for WT-NSUN2 versus 0.8% for ΔIDR-NSUN2) (Figure 4I). It is possible that the nucleoplasmic ΔIDR-NSUN2 preferentially methylates specific tRNA targets; a second possibility is that ΔIDR-NSUN2 methylates all tRNA targets less efficiently than WT-NSUN2.

**Figure 5.**
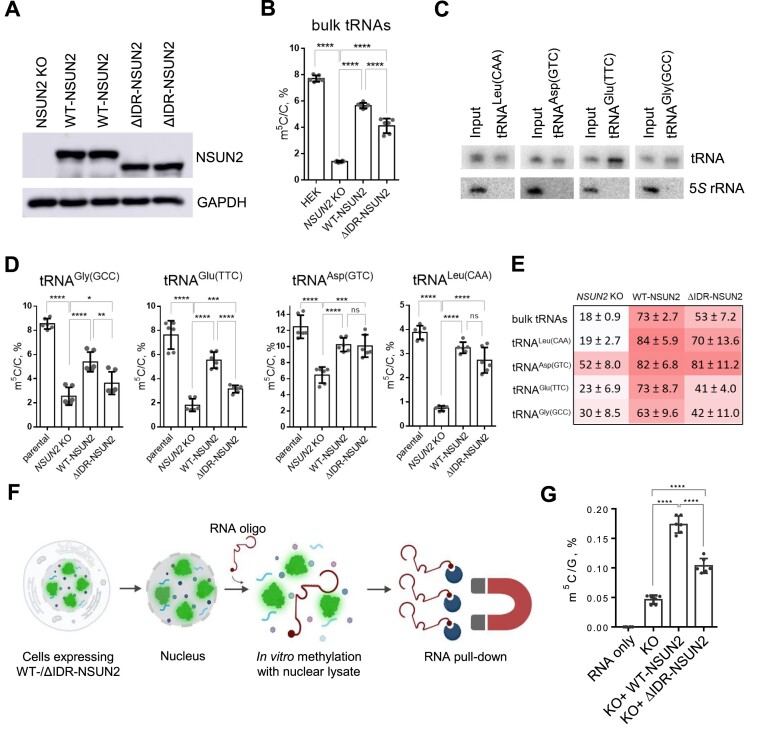
ΔIDR-NSUN2 restores m^5^C levels in specific tRNAs. (**A**) Western blots with anti-NSUN2 and anti-GAPDH antibodies showing the expression levels of mClover-WT-NSUN2 (WT-NSUN2) and mClover-ΔIDR-NSUN2 (ΔIDR-NSUN2), which have been transiently expressed in *NSUN2* KO cells. GAPDH serves as a loading control. (**B**) LC-MS/MS quantification of m^5^C levels in small RNA (<200 nt) used as input for tRNA purification in D. RNA were extracted from parental HEK293T cells, *NSUN2* KO cells and *NSUN2* KO transiently expressing mClover-WT-NSUN2 (WT-NSUN2) or mClover-ΔIDR-NSUN2 (ΔIDR-NSUN2). (**C**) Representative northern blot analysis showing enrichment of the target tRNAs (tRNA^Glu^, tRNA^Gly^, tRNA^Leu^ and tRNA^Asp^) after affinity purification. 5*S* rRNA serves as a negative control. (**D**) LC-MS/MS quantification of m^5^C levels of the purified tRNAs (tRNA^Glu^, tRNA^Gly^, tRNA^Leu^ and tRNA^Asp^) from parental HEK293T cells, *NSUN2* KO cells and *NSUN2* KO transiently expressing mClover-WT-NSUN2 (WT-NSUN2) or mClover-ΔIDR-NSUN2 (ΔIDR-NSUN2). Every sample is represented by three biological replicates, each was measured in two technical replicates. The two-tailored t-test was used to calculate the *P*-value (*P < 0.05, *** *P* < 0.001, **** *P* < 0.0001). Error bars represent mean ± S.D. (**E**) Heat map showing relative m^5^C level in bulk tRNA, tRNA^Glu^, tRNA^Gly^, tRNA^Leu^ and tRNA^Asp^ of parental HEK293T cells, *NSUN2* KO cells, and *NSUN2* KO transiently expressing mClover-WT-NSUN2 (WT-NSUN2) or mClover-ΔIDR-NSUN2 (ΔIDR-NSUN2). Values are normalized to the m^5^C level of parental cells, n = 6. (**F**) Schematic representation of *in vitro* methylation with nuclear extracts. Cells transiently expressing mClover-WT-NSUN2 or mClover-ΔIDR-NSUN2 were fractionated into cytoplasmic and nuclear fractions. Nuclear extracts were used for *in vitro* methylation on 5′biotynilated RNA oligo. After the reaction, biotinylated RNA probe was purified using magnetic streptavidin beads. (**G**) LC-MS/MS quantification of m^5^C levels of the purified RNA probe after *in vitro* methylation performed with the nuclear extracts prepared from *NSUN2* KO cells (KO), and *NSUN2* KO transiently expressing mClover-WT-NSUN2 (KO + WT-NSUN2) or mClover-ΔIDR-NSUN2 (KO + ΔIDR-NSUN2). Every sample is represented by three biological replicates, each was measured in two technical replicates. The two-tailored t-test was used to calculate the *p*-value (**** *p* < 0.0001). Error bars represent mean ± S.D.

To test these possibilities, we first modified the expression system by introducing the mClover tag to the N-terminus of NSUN2, allowing us to monitor the number of cells expressing mClover-NSUN2. Western blots show that the mClover-tagged WT-NSUN2 and ΔIDR-NSUN2 have comparable expression levels (Figure 5A, Supplementary Figure S6A–C). Expression of the mClover-WT-NSUN2 construct in more than 70% of the cells allowed us to rescue the m^5^C in bulk tRNAs to 5.7% (Figure 5B). Consistent with the previous results, despite similar expression levels (Figure 5A, Supplementary Figure S6A–C), bulk tRNA extracted from the mClover-WT-NSUN2 cells have significantly higher m^5^C levels compared to that of cells expressing mClover-ΔIDR-NSUN2 (5.7% versus 3.9%) (Figure 5B).

It has been shown that a proportion of NSUN2 localizes inside human mitochondria and is responsible for methylation of several mtRNAs (16,31). To investigate whether WT- and ΔIDR-NSUN2 have different ability to methylate mtRNAs, we separated cellular lysates into mitochondrial and cytoplasmic fractions after expression of mClover-WT-NSUN2 and mClover-ΔIDR-NSUN2 (Supplementary Figure S6D). Consistent with the previous studies, LC-MS/MS quantifications on mitochondrial small RNA (<200 nt) revealed significant reduction in m^5^C level in *NSUN2* KO cells compared to the control cells (Supplementary Figure S6E). Expression of mClover-WT-NSUN2 restored m^5^C levels in mtRNA from 0.12 ± 0.02 to 0.49 ± 0.10, while mClover-ΔIDR-NSUN2 restored m^5^C levels from 0.12 ± 0.02 to 0.33 ± 0.02. These data suggest that both WT- and ΔIDR-NSUN2 install m^5^C on mtRNAs; mtRNAs present different m^5^C levels between cells expressing WT- and ΔIDR-NSUN2 and the differences in m^5^C levels are comparable to those in bulk tRNAs.

Next, we quantified the m^5^C level of several specific NSUN2 tRNA targets. As NSUN2 methylates most of the cytoplasmic tRNA, we performed affinity purification of tRNA^Leu^, tRNA^Asp^, tRNA^Glu^ and tRNA^Gly^ using biotinylated complementary DNA oligos. We performed a Northern blot analysis to evaluate the efficiency of the purification. The results of the Northern blots, using the probes complementary to selected tRNAs and 5*S* rRNA as a control, demonstrate successful enrichment of the tRNAs in the pull-down samples (Figure 5C). LC-MS/MS analysis of the purified tRNAs revealed that all the selected tRNA targets were partially methylated by both mClover-WT-NSUN2 and mClover-ΔIDR-NSUN2 (Figure 5D and E). The methylation status of the different tRNAs varied after expression of mClover-WT-NSUN2: m^5^C levels of tRNA^Leu^ and tRNA^Asp^ were almost completely restored (to 84% for tRNA^Leu^ and 83% for tRNA^Asp^), while m^5^C levels of tRNA^Glu^ and tRNA^Gly^ were only restored to 73% and 63%, respectively. Notably, methylation levels of tRNA^Leu^, tRNA^Glu^ and tRNA^Gly^ after mClover-ΔIDR-NSUN2 expression were significantly lower than the methylation levels of the same tRNAs after expression of mClover-WT-NSUN2 (70% versus 84% for tRNA^Leu^: 41% versus 73% for tRNA^Glu^ and 63% versus 72% for tRNA^Gly^). Interestingly, tRNA^Asp^, unlike other tested tRNA targets, was almost fully methylated after the expression of both mClover-WT-NSUN2 and mClover-ΔIDR-NSUN2 expression. Taken together, the data suggest that while certain tRNAs are methylated to a similar extent by both WT- and ΔIDR-NSUN2, nucleoplasm-localized ΔIDR-NSUN2 has a deficiency in installing m^5^C on specific tRNAs.

To elucidate the contribution of nucleoplasmic localization into moderate catalytic activity of ΔIDR-NSUN2 on specific tRNA targets, we utilized *in vitro* methylation assays. Since recombinant ΔIDR-NSUN2 was unstable, we performed *in vitro* methylation assay using nuclear extract prepared from *NSUN2* KO HEK293T cells transiently expressing mClover-WT-NSUN2 or mClover-ΔIDR-NSUN2 on biotinylated RNA oligo resembling part of tRNA^Gly^ (Figure 5F). Despite the fact that utilized nuclear extracts had comparable concentration of WT- and ΔIDR-NSUN2 (Supplementary Figure S6F), extracts containing ΔIDR-NSUN2 showed significantly lower m^5^C methylation activity compared to the extracts containing WT-NSUN2 (Figure 5G). These data suggest that the difference in m^5^C methylation levels of specific tRNAs between cells expressing WT- and ΔIDR-NSUN2 is likely determined by the impaired catalytical activity of ΔIDR-NSUN2 rather than the nucleoplasm localization of ΔIDR-NSUN2, which may explain the differences in the cognitive recognition between WT and ΔIDR-NSUN2-expressing *Drosophila*.

## Discussion

**Figure 6.**
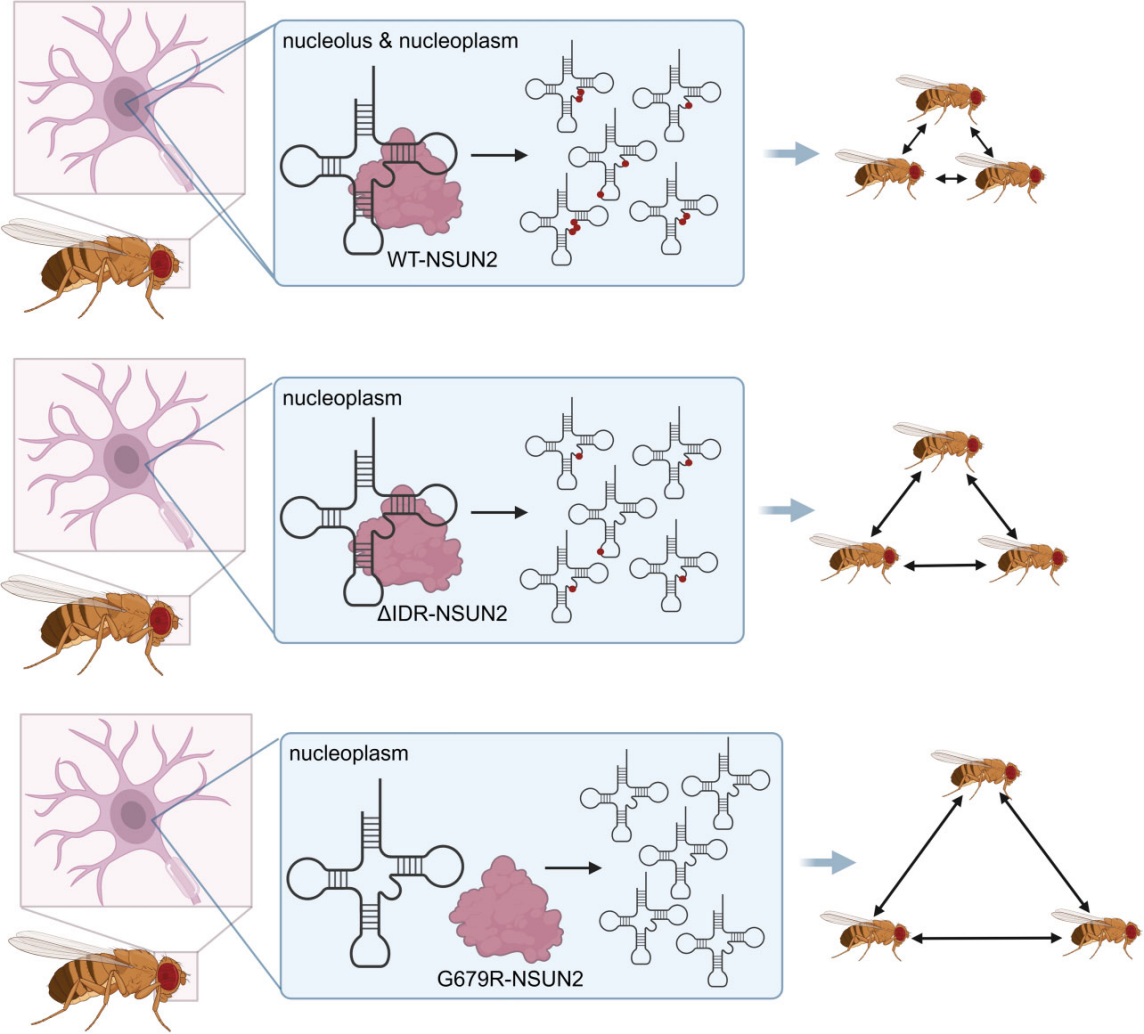
NSUN2-mediated m^5^C installation is indispensable for proper social behavior in *Drosophila*. Schematic illustrating how WT-NSUN2 and NSUN2 mutants install m^5^C in tRNAs, leading to distinct impacts on social behavior in *Drosophila melanogaster*. Created in BioRender. Upenn, L. (2024) https://BioRender.com/s35c630.

Dysregulation of tRNA-modifying enzymes is frequently implicated in human diseases including neurological disorders (39). SAM-dependent methyltransferase NSUN2, which installs m^5^C in tRNAs, has been previously shown to be critical in neurodevelopment in several organisms (14,22,35). Besides several nonsense mutations, one G679R missense mutation of NSUN2 causes intellectual disabilities in humans. It was shown that the G679R mutation leads to the mislocalization of NSUN2 from the nucleolus to the nucleoplasm. However, it was unknown whether G679R causes NSUN2 catalytic deficiency or if nucleolar localization is detrimental to tRNA methylation. Here we show that the G679R mutation reduces NSUN2’s tRNA-binding affinity and thus causes tRNA m^5^C hypomethylation. This study also suggests the nucleolar localization of NSUN2 may not be essential for the installation and function of tRNA m^5^C (Figure 6).

The cellular localization of m^5^C installation on tRNA was first reported in a study where mouse fibroblast lysates were separated into nuclear and cytoplasmic fractions to measure their tRNA methyltransferase activity (40). These *in vitro* methylation experiments showed that the nuclear extracts had the main m^5^C installing activity (40). Subsequently, immunostaining analysis revealed that NSUN2, the enzyme that mediates tRNA m^5^C installation, localizes to the nucleolus (17). It was also suggested that NSUN2-specific methylation of tRNAs was inhibited by the nucleolar exclusion of NSUN2 after UV radiation and arsenate treatment, as a response to cellular stress (14). In this study, we designed a truncation variant of NSUN2, ΔIDR-NSUN2, that is excluded from the nucleolus and localizes to the nucleoplasm. We expressed ΔIDR-NSUN2 in *NSUN2* KO cells and observed a significant increase in m^5^C level in the tRNA pool (Figure 2), indicating that NSUN2-mediated m^5^C installation is not restricted to the nucleolus and can occur in the nucleoplasm.

Interestingly, the m^5^C level in tRNAs after expressing WT- and ΔIDR-NSUN2 exhibited a small yet significant difference. The expression of ΔIDR-NSUN2 consistently resulted in ∼20% lower m^5^C level in bulk tRNAs compared to the expression of WT-NSUN2. Measurement of the m^5^C level of individual NSUN2 targets demonstrated discrepancy in the m^5^C level of different tRNAs. While tRNA^Asp^ and tRNA^Leu^ were methylated to a similar extent by WT- and ΔIDR-NSUN2, tRNA^Gly^ and tRNA^Glu^ carried ∼64% less m^5^C modifications after ΔIDR-NSUN2 expression compared to the expression of WT-NSUN2 (Figure 5D and E). The *in vitro* methylation experiments with the nuclear extracts demonstrated that ΔIDR-NSUN2 expose weaker catalytic activity compare to WT-NSUN2 and the detected difference in the m^5^C methylation status of tRNA^Gly^ and tRNA^Glu^ can not be explained by cellular localization of ΔIDR-NSUN2 (Figure 5G). This interesting phenomenon can be attributed either (i) to the difference in turnover of the selected tRNAs or (ii) specific preferences of ΔIDR-NSUN2 in installing m^5^C methylation on the selected tRNA substrates determined by the nature of the truncation.

To evaluate the pathological impact of G679R-NSUN2 on neurological function, we utilized *Drosophila melanogaster* as the model organism. Consistent with other studies of NSUN2 in complex organisms (21,22), deletion of *Nsun2* was not essential for the survival of the fruit flies. Previously, Abassi and colleagues determined the cognitive phenotype of the *Nsun2-*deficient flies by assessing their learning abilities. In behavioral experiments, *Nsun2* KO flies exhibited a lack of olfactory short-term memory, and expressing NSUN2 in neurons fully rescued the observed phenotype (22). In our study, we analyzed the social behavior of *Nsun2* KO flies by assessing their social space and the distance in which flies position themselves relative to each other. We found that *Nsun2* KO caused impairments in social interactions of the flies (Figure 4). Interestingly, immunostaining of the mushroom body, the brain structure implicated in insect learning, memory, and social behavior (41), did not reveal obvious changes upon *Nsun2* KO (Supplementary Figure S5C), suggesting that loss of Nsun2 does not affect gross development of the nervous system, but rather the cognitive function. Notably, LC-MS/MS analysis of small RNAs revealed a drastic difference in the m^5^C level of tRNA pools isolated from the whole flies (3.4% ± 0.07) and fly heads (7.1% ± 0.75), emphasizing the importance of the NSUN2-mediated m^5^C modification in tRNAs for the nervous system (Figure 4D). Expression of human WT- and ΔIDR-NSUN2 in neurons of *Nsun2* KO flies increased m^5^C level in tRNAs and partially restored the social behavioral phenotype in contrast to G679R-NSUN2, which failed to increase m^5^C level and influence social behavior pattern (Figure 6).

Collectively, this work reveals the molecular-level mechanism underlying the NSUN2 disease variant G679R in causing intellectual disability, comprehends the spatial regulation and biogenesis of tRNA modifications and extends our knowledge of the biological impacts of NSUN2-mediated tRNA methylation in cognition function.

## Supplementary Material

gkae1169_Supplemental_File

## Data Availability

All the materials and methods have been provided in the main text and supplemental information. Further information and requests for resources and reagents should be directed to and will be fulfilled by the corresponding author, Kathy Fange Liu (liufg@pennmedicine.upenn.edu).
