# Supplementary material for: Spatial regulation of NSUN2-mediated tRNA m5C installation in cognitive function
Source: Nucleic Acids Res. 2024 Dec 14;53(2):gkae1169. doi: 10.1093/nar/gkae1169 (PMC11754655; doi:10.1093/nar/gkae1169)
Supplement: gkae1169_Supplemental_File [file gkae1169_supplemental_file.docx]

**Supporting information**

**Spatial Regulation of NSUN2-mediated tRNA m^5^C installation in Cognitive Function**

**AUTHORS**

Yulia Gonskikh^1^, Christian Tirrito^2,3^, Praneeth Bommisetti^1^, Sarai Mendoza^1^, Julian Stoute^1^, Joshua Kim^3^, Qin Wang^3^, Yuanquan Song^3,4,^* and Kathy Fange Liu^1,5,6,7,8,^*

^1^Department of Biochemistry and Biophysics, Perelman School of Medicine, University of Pennsylvania, Philadelphia, PA 19104, USA

^2^Biology Graduate Group, University of Pennsylvania, School of Arts and Sciences, Philadelphia, PA 19104, USA

^3^The Raymond G. Perelman Center for Cellular and Molecular Therapeutics, The Children’s Hospital of Philadelphia, Philadelphia, PA 19104, USA

^4^Department of Pathology and Laboratory Medicine, University of Pennsylvania, Philadelphia, PA 19104, USA

^5^Graduate Group in Biochemistry and Molecular Biophysics, Perelman School of Medicine, University of Pennsylvania, Philadelphia, PA 19104, USA

^6^Abramson Family Cancer Research Institute, University of Pennsylvania Perelman School of Medicine, Philadelphia, PA 19104, USA

^7^Penn Institute for RNA Innovation, University of Pennsylvania, Philadelphia, PA 19104, USA

^8^Penn Center for Genome Integrity, University of Pennsylvania, Philadelphia, PA 19104, USA

*Correspondence to [liufg@pennmedicine.upenn.edu](mailto:liufg@pennmedicine.upenn.edu), songy2@chop.edu

**
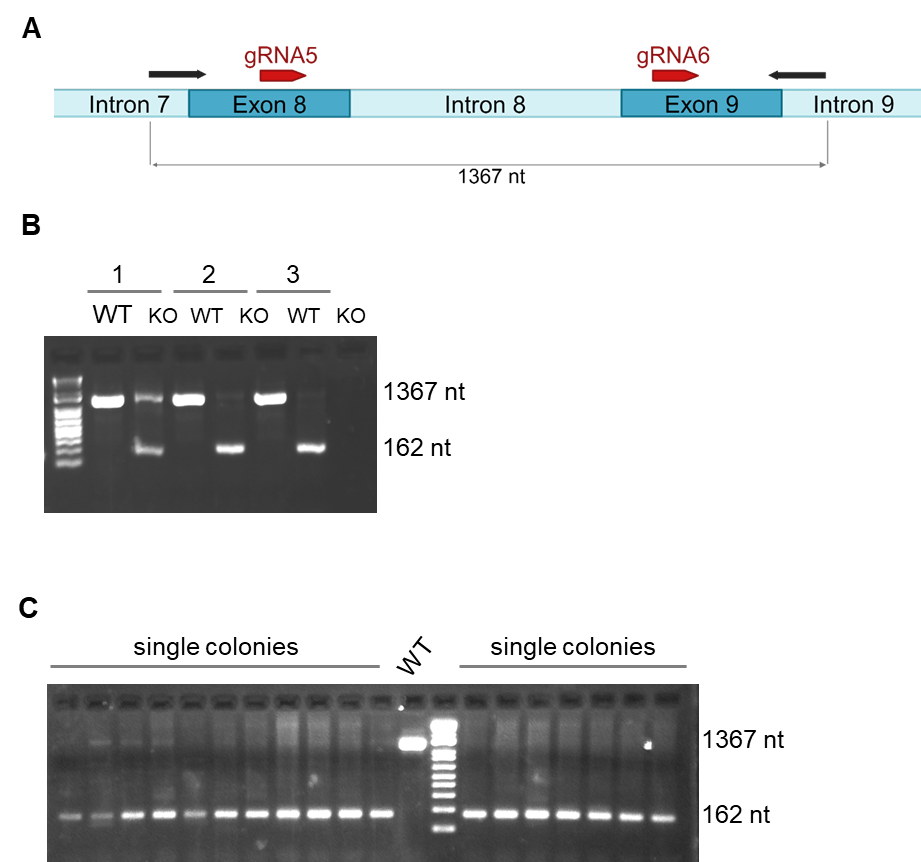
**

**Supplementary Figure S1. Construction of *NSUN2* knockout HEK293T cells.** (**A**) Schematic representation of using sgRNA to target NSUN2 gene. sgRNA5 and sgRNA6, depicted by red arrows, target exon 8 and exon 9, respectively. Primers used in the validation PCR are shown by black arrows. (**B**) The validation PCR results are shown. The primers shown in (**A**) were used to PCR with the genomic DNA isolated from the bulk cell population before single-cell sorting. (**C**) PCR screening on the genomic DNA isolated from single cell-derived clones with the primers shown in (**A**).

**
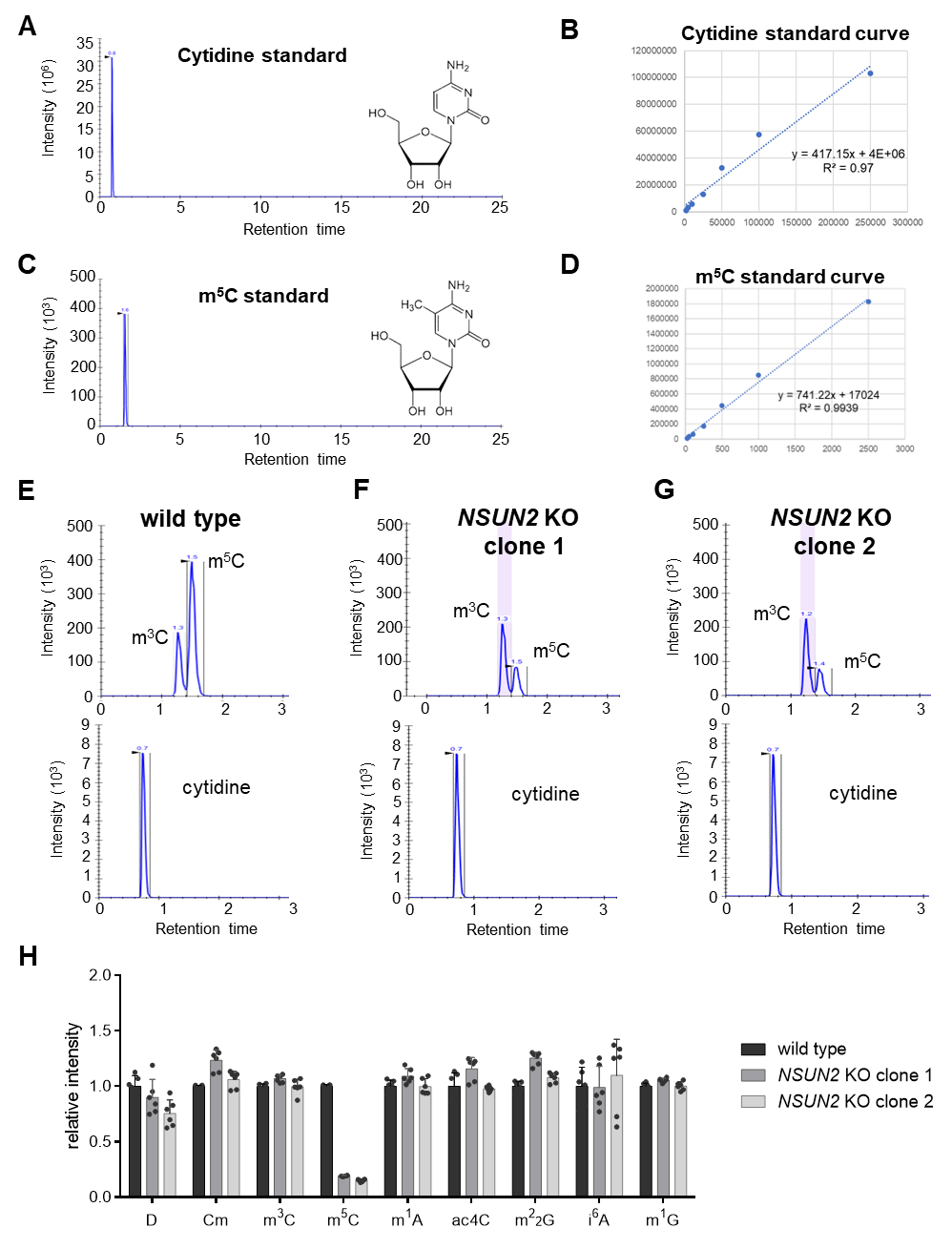
**

**Supplementary Figure S2. Modifications levels of small RNAs (< 200 nt) in *NSUN2* knockout cells.** (**A**) LC-MS/MS channel and peak area of cytidine. (**B**) LC-MS/MS quantification standard curves of cytidine. (**C**) LC-MS/MS channel and peak area of m^5^C. (**D**) LC-MS/MS quantification standard curves of m^5^C. (**F**-**G**) LC-MS/MS channel and peak area of m^3^C, m^5^C, and cytidine in (**E**) wild-type and (**F** – **G**) two single cell-derived *NSUN2* knockout clones. (**H**) LC-MS/MS quantification of tRNA modifications in < 200 nt RNAs isolated from wild type and *NSUN2* KO clones. The mean of the abundance of each modification in wild type samples was taken as one. Every sample is represented by 3 biological replicates, each was measured in two technical replicates.

**
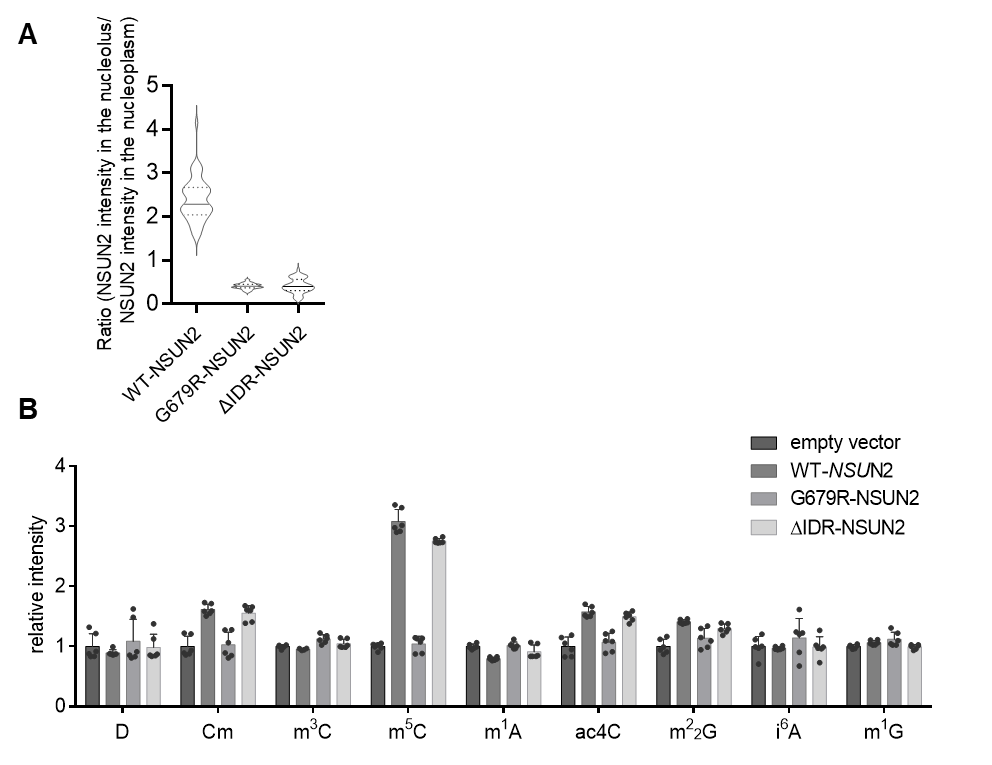
**

**Supplementary Figure S3. Immunostaining and LC-MS/MS analysis after transient expression of NSUN2.** (**A**) Relative intensity of transiently expressed WT-, G679R-, or ΔIDR-NSUN2 in the nucleolus of *NSUN2* KO HEK293T cells. Intensity of WT-, G679R-, or ΔIDR-NSUN2 in the nucleolus was normalized to its intensity in the nucleoplasm of the same cell. Every sample is represented by 50 measurements. (**B**) LC-MS/MS quantification of tRNA modifications in < 200 nt RNAs isolated from *NSUN2* KO cells transiently expressing empty vector, WT-, G679R-, or ΔIDR-NSUN2. The mean of the abundance of each modification in empty vector samples was taken as one. Every sample is represented by 3 biological replicates, each was measured in two technical replicates.

**
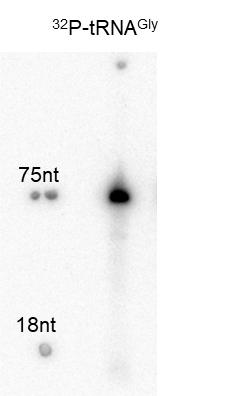
**

**Figure S4. *In vitro* transcription of tRNA^Gly^.** Autoradiogram of *in vitro* transcribed ^32^P-tRNA^Gly^ on polyacrylamide denaturing gel. 75 and 18 nucleotides markers are indicated.


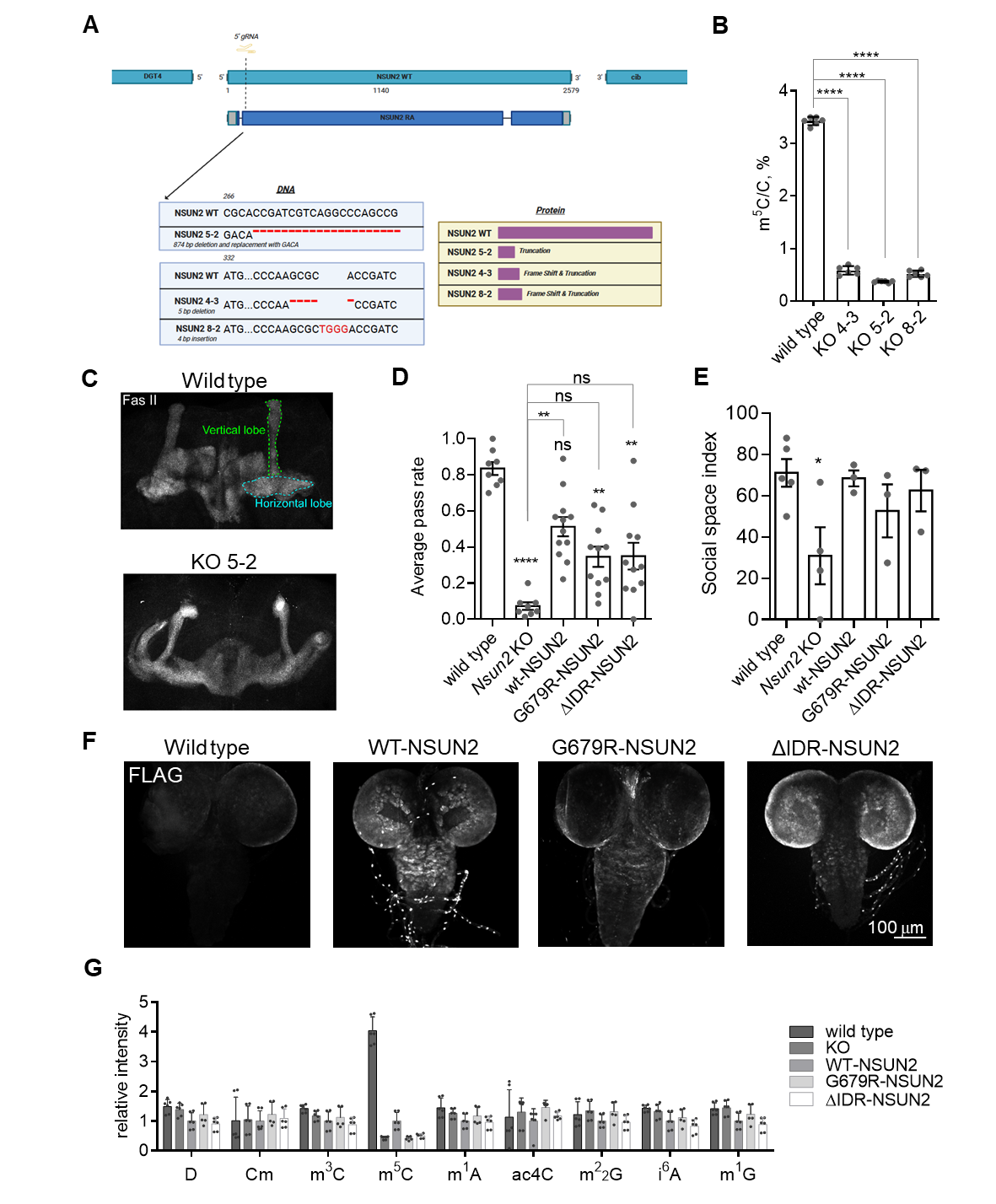


**Supplementary Figure S5. Importance of NSUN2-mediated m^5^C for cognitive function in *Drosophila melanogaster*.** (**A**) Schematic representation of sgRNA to target *Nsun2* gene in *Drosophila* and the alleles generated. (**B**) LC-MS/MS quantification of m^5^C in small RNA (< 200 nt) isolated from wild-type or *Nsun2* KO flies. (**C**) Representative immunostaining analysis of adult fly brains stained with anti-FasII antibody. Vertical and horizontal lobes are indicated. (**D**) The negative geotaxis assay shows that *Nsun2* KO drastically reduces the percentage of flies passing a set threshold, which is significantly rescued only by pan-neural overexpression of WT-NSUN2. n= 8-12 groups. Data are analyzed by one-way ANOVA followed by Dunn’s test. Each group is compared to every other group. Compared to wild type, *NSUN2 KO*, G679R-NSUN2, ΔIDR-NSUN2 are significantly reduced, but not wt-NSUN2. Compared to *NSUN2 KO*, wt-NSUN2 significantly rescues the phenotype, but not G679R-NSUN2 or ΔIDR-NSUN2. (**E**) *Nsun2* KO flies show reduced social space index. Data are analyzed by one-way ANOVA followed by Dunn’s test. n= 3-5 groups. **p* < 0.05, ****p* < 0.001, *****p* < 0.0001. ns: not significant. (**F**) Immunostaining of dissected fly larval brain confirms the expression of NSUN2 transgenes. Scale bar = 100 μm. (**G**) LC-MS/MS quantification of tRNA modifications in < 200 nt RNAs isolated from flies’ heads of wild type, Nsun2 KO, and NSUN2 KO flies expressing WT-, G679R-, and ΔIDR-NSUN2. The mean of the abundance of each modification in wild type samples was taken as one. Every sample is represented by 3 biological replicates, each was measured in two technical replicates.

**
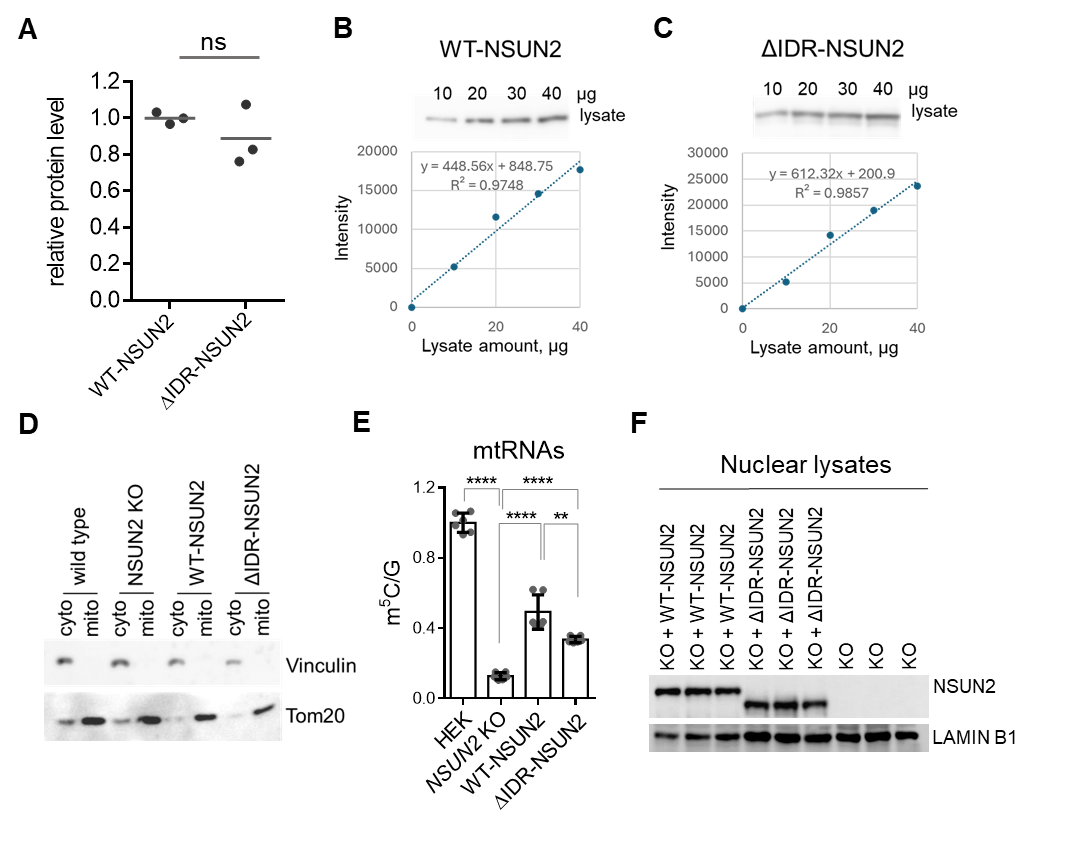
Supplementary Figure S6. Effect of WT- and ΔIDR-NSUN2 expression m^5^C levels in tRNAs.** (**A**) Quantification of western blots showing transient expression of WT- and ΔIDR-NSUN2 in lysates used for extraction of small RNA analyzed by LC-MS/MS analysis. Each measurement represents biological replicate. Values were normalized to the mean of the WT-NSUN2 protein intensity. ns stands for not significant. (**B** and **C**) Western blot analysis with anti-NSUN2 antibody on 10, 20, 30, and 40 µg lysate expressing WT- or ΔIDR-NSUN2 and corresponding western blot standard curves. (**D**) Representative western blot analysis with anti-Vinculin (cytoplasmic marker) and anti-Tom20 (mitochondrial marker) showing cellular fractionation into cytoplasmic (cyto) and mitochondrial (mito) fractions, performed on wild type cells, NSUN2 KO cells, and NSUN2 KO cells transiently expressing WT- and ΔIDR-NSUN2. (**E**) LC-MS/MS quantification of m^5^C in < 200 nt mitochondrial RNAs isolated of *NSUN2* KO cells transiently expressing empty vector, WT-NSUN2, and ΔIDR-NSUN2. Every sample is represented by 3 biological replicates; each was measured in two technical replicates. The two-tailored *t*-test was used to calculate the *p*-value (****p < 0.0001, **p = 0.0032). (**F**) Representative western blot analysis with anti-NSUN2 and anti-Lamin B1 antibody performed on nuclear extracts prepared from NSUN2 KO cells, and NSUN2 KO cells transiently expressing WT- and ΔIDR-NSUN2. Each sample represented by 3 biological replicates.

**Supplementary Table S1. DNA and RNA oligos used in this study.**

| **name** | **Sequence (5’ to 3’)** |
| --- | --- |
| gRNA5 | UAGCUGCAAGCUAUUUAAGG |
| gRNA5_for | CACCGTAGCTGCAAGCTATTTAAGG |
| gRNA5_rev | AAACCCTTAAATAGCTTGCAGCTAC |
| gRNA6 | AAUAGGGUUUAGUGAACACG |
| gRNA6_for | CACCGAATAGGGTTTAGTGAACACG |
| gRNA6_rev | AAACCGTGTTCACTAAACCCTATTC |
| hNSUN2_KO5-6_for | ACCTGTAGGGAGCTTTGCAC |
| hNSUN2_KO5-6_rev | TCCATCAAGCGTGCTTACCTT |
| NSUN2_G679R_for | GCAGATGGCGGGGAAAGGCCTCCATTC |
| NSUN2_G679R_rev | CCATCTGCATAAGACGATGGGACACTGC |
| NSUN2_ΔIDR_for | TGTGGCAGTATTGGTGAAAGTGTGTGGTCCTCCTCCATC |
| NSUN2_ΔIDR_rev | GATGGAGGAGGACCACACACTTTCACCAATACTGCCACA |
| NSUN2_Mfe1_for | AAATTTCAATTGATGGGGCGGCGGTCGCGGGGTC |
| NSUN2_Xho1_rev | GACACTCGAGTCACCGGGGTGGATGGACCCCCG |
| NSUN2_TEV_EcoRI_for | CGGAATTCGAAAACCTGTACTTCCAGGGAATGGGGCGGCGGTCGCGGGGTC |
| NSUN2_6His_SalI_rev. | ACGCGTCGACTTAGTGGTGATGGTGATGATGTCCCCGGGGTGGATGGACCCCCG |
| PPB_XhoI_for | CATACTCGAGATGGACTACAAAGACGATGACGACAAG |
| NSUN2_XbaI_rev | CATATTCTAGAATTCCTCGAGTCACCGGGGT |
| RNA for *in vitro* methylation (modifications sites are underlined) | CUGCCACGCGGGAGGCCCGGGUUCGAUU    Biotin-CUGCCACGCGGGAGGCCCGGGUUCGAUU |
| tRNA_Gly_for | GAGTAATACGACTCACTATAGGATTGGTGGTTCAGTGGTAGAATTCTCGCCTGCCACGCGGGAG |
| tRNA_Gly_rev | ATTGTCGGATCCTGGTGGATTGGCCGGGAATCGAACCCGGGCCTCCCGCGTGGCAGGCGAGA |
| tRNA_Gly_for2 | GACAATCTCGAGTAATACGACTCACTATA |
| tRNA_Gly_rev2 | ATTGTCGGATCCTGGTGGATT |
| tRNAPD_Leu CAA | Biotin-CGATTCGAACCCACGCCTCCA |
| tRNAPD_Gly GCC | Biotin-TGCATTGGCCGGGAATCGAACCCGGGCCTC |
| tRNAPD_Asp GUC | Biotin-CTCCCCGTCGGGGAATCGAACCCCGGTCTC |
| tRNAPD_Glu UUC | Biotin-TTCCCACACCGGGAGTCGAACCCGGGCCGCCT |
